# Response of Potato Tuber Number and Spatial Distribution to Plant Density in Different Growing Seasons in Southwest China

**DOI:** 10.3389/fpls.2016.00365

**Published:** 2016-04-08

**Authors:** Shun-Lin Zheng, Liang-Jun Wang, Nian-Xin Wan, Lei Zhong, Shao-Meng Zhou, Wei He, Ji-Chao Yuan

**Affiliations:** ^1^Agronomy, Sichuan Agricultural UniversityChengdu, China; ^2^Agriculture Bureau of Dongpo County, Agrotechnical StationMeishan, China; ^3^Sichuan Academy of Agricultural Sciences, Crop Research InstituteChengdu, China

**Keywords:** potato, growing season, plant density, tuber yield, spatial distribution, artificial neural network model

## Abstract

The aim of this study was to explore the effects of different density treatments on potato spatial distribution and yield in spring and fall. Plant density influenced yield and composition, horizontal, and vertical distribution distances between potato tubers, and spatial distribution position of tuber weights. The results indicated that: (1) Spring potato yield had a convex quadratic curve relationship with density, and the highest value was observed at 15.75 × 10^4^ tubers per hectare. However, the yield of fall potatoes showed a linear relationship with plant density, and the highest value was observed at 18 × 10^4^ tubers per hectare; (2) Density had a greater influence on the tuber weight of spring potatoes and fruit number of single fall potatoes; (3) The number of potato tubers in the longitudinal concentration exhibited a negative linear relationship with density, whereas the average vertical distribution distance of tubers exhibited a positive incremental hyperbolic relationship. For spring and fall potato tubers, the maximum distances were 8.4152 and 6.3316 cm, and the minimum distances 8.7666 and 6.9366 cm, respectively; and (4) Based on the artificial neural network model of the spatial distribution of tuber weight, density mainly affected the number and spatial distribution of tubers over 80 g. Tubers over 80 g were mainly distributed longitudinally (6–10 cm) and transversely (12–20 cm) within the high density treatment, and the transverse distribution scope and number of tubers over 80 g were reduced significantly. Spring potato tubers over 80 g grown at the lowest density were mainly distributed between 12 and 20 cm, whereas those at the highest density were primarily distributed between 10 and 15 cm.

## Introduction

With the increase in China's population and decrease in arable land, food security issues have become more prominent. Potato (*Solanum tuberosum* L.) is one of the most important staple food crops in China and plays important roles in coping with multiple crop indices, the output of cultivated land, and food security problems (Seyed and Asghar, 2011). In southwestern China, potatoes are planted in the spring and fall, leading to a relative high land output. The average potato yield in China is ~1.5 × 104 kg hm^−2^, whereas the average global yield is about 1.7 × 104 kg hm^−2^. Theoretically, yield can reach 12 × 104 kg hm^−2^, revealing that there is potential to further improve potato production in China (Qu et al., 2005; Jia et al., 2011).

Increase in plant density is an effective way to improve potato yield, but certain differences exist among high densities and growing seasons (Vasilyev, 2014). Yu et al. (2009) showed that fall potato yield is positively correlated with density. Zhao et al. (2005) found that plant density has a downward parabolic relationship with potato yield, and delayed sowing dates require increased densities to obtain the maximum yield. Agricultural methods in China have become more mechanized in order to achieve higher efficiency and increase potato yields, but the rate of tuber injury during potato harvest is high. For instance, previous studies indicated that 70% of potato injuries are caused during harvest, and that the injury rates associated with potato harvest are ~30% of the total output (Peters, 1996; Wang et al., 2014). These factors seriously influence the yield and commodity value of potato.

The design used to harvest potatoes is based on the horizontal and vertical distribution of tubers as well as the sowing depth (Zhang, 2014). Several factors affect the potato tuber distribution (Wurrt et al., 1993). For instance, stolon plays a decisive role in the size and distribution of tubers, and larger leaf areas and the accumulation of leaf dry matter are beneficial to the stolon formation (Liu et al., 2003). Moreover, larger canopy sizes can significantly promote tuber expansion (Yang et al., 1994). Previous studies have confirmed that the leaf area index (LAI) increases with the increasing plant density (Jin et al., 2013). However, high densities result in both decreased leaf area and photosynthetic rates. Furthermore, the crown and stolon number per plant increases with the increasing density, whereas the average potato weight decreases significantly (Xiao et al., 2003; Fu, 2012). These results implied that plant density increase results in individual competition, and individual growth inhibition eventually leads to differences in the spatial distribution of potato tubers. Previous studies on potato have focused on analyzing tuber size and number (Haverkort et al., 1990; Wurrt et al., 1997); however, little is known about the impact of planting density on tuber spatial distribution.

To address these issues, the present study aimed to investigate the differences between potato crops grown at different densities in spring and fall. In addition, this study investigated the spring and fall potato yield, the spatial distribution of tubers, and the relationship between density and tuber spatial distribution. The results of this study will provide information on the appropriate densities for mechanical harvest that increase potato yield.

## Materials and methods

### Site description

The study was conducted at the experimental farm of Southwest Sichuan Agricultural University, Chengdu, Sichuan Province, southwest China (N30°67′, E 104°06′). Soil and weather data are shown in Tables 1, 2, respectively.

**Table 1:** **Soil conditions in the two experimental sites**.

**Experimental site**	**Soil type**	**pH**	**Organic matter (g kg^−1^)**	**Total N content (g kg^−1^)**	**Total P content (g kg^−1^)**	**Total K content (g kg^−1^)**	**Available N (mg kg^−1^)**	**Available P (mg kg^−1^)**	**Available K (mg kg^−1^)**
Spring site	PS	5.09	25.09	1.98	0.83	14.20	136.82	163.33	107.00
Fall site	PS	5.92	24.54	1.85	0.92	14.12	191.94	126.99	91.29

*PS, paddy soil; Soil type in both seasons was consistent, and the experimental sites were previously planted with rice*.

**Table 2:** **Meteorological factors at each growing stage of spring and fall potatoes**.

**Growing season**	**Growth stage**	**Rain (mm)**	**AT (≥5 °C)**	**SD (h)**	**Day length (h)**	**DMT (°C)**
	SS-MS	139.0	1381.5	332.9	1025.03	17.3
Spring	SS-TBS	7.9	513.0	149.9	411.45	15.5
	TBS-MS	131.1	868.5	183.0	613.58	18.5
	SS-MS	209.3	1239.7	146.7	873.20	16.1
Fall	SS-TBS	165.6	594.6	70.0	346.43	20.5
	TBS-MS	43.7	645.1	76.7	526.77	13.4

### Experimental materials and design

Potato tubers (*S. tuberosum* cv. Chuanyu 117) were provided by the Crops Institute, Sichuan Academy of Agricultural Sciences, China. The study was conducted using the following plant densities: D1 (6 × 10^4^ strains hm^−2^), D2 (9 × 10^4^ strains hm^−2^), D3 (12 × 10^4^ strains hm^−2^), D4 (15 × 10^4^ strains hm^−2^), and D5 (18 × 10^4^ strains hm^−2^). A randomized block design with three replications was used in two growing seasons, spring and fall. The plot area was 14 m^2^ (2 m × 7 m) with a 60 or 40-cm row space. Whole tubers (~30–40 g) were planted at a depth of ~10 cm. The amount of compound fertilizer used was ~127.5 kg hm^−2^, and field management was according to local practices. Irrigation was applied to maintain moisture at field capacity. Spring potato plants were harvested 5 months after sowing in December 2012, and fall potato plants were harvested 4 months after sowing in August 2013.

### Sampling and determination of variables

The effective plant number was estimated based on the actual number plants at 14 days post emergence, and the effective unit area number based on the germination rate. Ten representative mature plants were selected from each plot to determine tuber weight, stems at the ridge surface level of mutilation, stem center, and ridge surface at the water level under different densities and position distribution models. The transverse distribution of the vertical tuber level was estimated from the stem to the furthest vertical distance, whereas the longitudinal distribution distance from the stem to the furthest horizontal distance at the bottom of the ridge surface to the tuber. The tuber weight was measured using an electronic scale. Twenty representative mature plants were selected from each experimental plot to determine yield and yield components under different densities and position distribution models. All statistical analyses were performed using Excel (Microsoft Corp., Redmond, WA, USA), Alphatruck 2.0 (Middlesex, UK), Sigmaplot 12.5 (Softonic International, Barcelona, Spain), and JMP 10 (SAS, Cary, NC, USA).

## Results

### Influence of density on yield and yield components

Tuber number and tuber weight significantly decreased with the increasing plant density increased (Table 3), whereas the effective plant number increased. Differences between potato plant growing seasons and the effects of density on yield varied significantly. The variable coefficients of yield, fruit number, and tuber weight were 5.80, 13.18, and 28.34% for single spring potatoes, respectively, and 11.22, 17.85, and 11.22% for single fall potatoes, respectively. Spring potato tuber weight was significantly influenced by plant density, whereas fall potato tuber weight by single potato fruit number.

**Table 3:** **Yield and yield components under different plant densities in the two growing seasons**.

**Growing season**	**Density (× 10^4^ plant·hm^−2^)**	**Yield (thm^−2^)**	**Effective plants (× 10^4^ plant hm^−2^)**	**Tubers plant^−1^**	**G tuber^−1^**
Spring	6	42.05c	5.52e	7.82a	103.22a
	9	44.27b	8.20d	7.75ab	73.87b
	12	47.71a	11.00c	7.00b	65.60c
	15	48.05a	13.94b	5.87c	62.18d
	18	47.63a	16.41a	6.08c	49.53e
Average		45.94	11.01	6.90	70.88
Fall	6	21.29d	5.84e	5.67a	62.77a
	9	22.52cd	8.73d	4.27b	56.53ab
	12	23.95bc	11.18c	4.40b	50.36b
	15	25.91b	13.65b	3.67b	54.75b
	18	28.17a	15.42a	3.87b	51.26b
Average		24.37	10.97	4.38	55.13

*Data are presented as means of three replicates in each treatment. Different letters in each column represent significant differences at p < 0.05*.

Spring potato yield and plant density exhibited a quadratic function relationship and the regression equation was: *y* = 0.0665*x*^2^+2.0942+31.5860 (*R*^2^ = 0.9638^*^). The highest yield was measured at 15.75 × 10^4^ tubers or plants hm^−2^. However, fall potato yield and plant density exhibited a linear relationship, and the regression equation was: *y* = 0.5717*x*+17.5080 (*R*^2^ = 0.9838^**^). The highest yield was measured of 0.5717 × 10^3^ kg hm^−2^ was measured at 1.00 × 10^4^ tubers or plants·hm^−2^.

Correlation analysis (Table 4) revealed that the effective plant number was positively associated with yield, whereas the tuber number and weight values were negatively associated. No significant correlations were found between fall potato tuber weight and yield among plant densities. Furthermore, based on size, analysis showed that the effective plant number mostly affected yield, but the yield components of the two growing seasons differed slightly in the contribution rate. For spring potatoes, the contribution of weight was higher than that of potato number or effective strains, whereas for fall potatoes, the contribution of the effective plant number was higher than that of potato number or potato weight. During spring, the increased density maintained large tuber weights, and the increased density during fall mainly increased yield.

**Table 4:** **Contribution of yield components to yield**.

**Growing season**	**Yield components**	**Correlation coefficient**	**Direct effect**	**Contribution rate (%)**
Spring	Effective plants	0.8484^**^	−0.1791	14.15
	Tuber number per plant	−0.7567^**^	−0.3408	24.02
	Single tuber weight	−0.8602^**^	−0.7717	61.83
	Effective plants	0.8946^**^	1.4000	77.20
Fall	Tuber number per plant	−0.7091^**^	0.2828	12.36
	Single tuber weight	−0.4511	0.3755	10.44

^**^; stands for each index contributed to yield reached extremely significant level.

### The relationship between density and average distribution of potato tuber distance

A significant difference in potato tuber distribution was observed (Table 5), and the average distance transverse distribution coefficients of variation were 6.38 and 4.11% for spring and fall potatoes, respectively. The influence of density on the longitudinal distribution of the average distance of potato tubers was relatively high. Moreover, the average distance of transverse distribution was essentially the same, whereas the effect of the transverse distribution on the average distance was greater. A general relationship between the average distribution of the longitudinal distance (*Z*) and density (*x*) fit well with the incremental hyperbolic function (*Z* = *a*+*b*∕*x*). The equations were *Z* = −9.8196∕*x*+8.4152 (*R*^2^ = 0.8358^*^) for spring and *Z* = −5.2716∕*x*+6.3316 (*R*^2^ = 0.9547^^**^;^) for fall. The equations suggested that the maximum distance between the vertical distribution of spring and fall potato tubers was on average 8.4152 and 6.3316 cm, respectively. The transverse distribution of the average distance (*H*) and density (*x*) were positively related to the decline of the hyperbolic function (*H* = *a*+*b*∕*x*), and the equations were *H* = 29.3939∕*x*+29.3939 (*R*^2^ = 0.9638^^**^;^) for spring and *H* = 22.0697∕*x*+22.0697 (*R*^2^ = 0.9670^^**^;^) for fall. These equations showed that the minimum transverse distribution of the average distance of spring and fall potato tubers was 8.7666 and 6.9366 cm, respectively. Data indicated that the transverse distribution range was larger than the longitudinal distance of potato tubers. Furthermore, the transverse and vertical distance of spring potato tubers was larger than that of fall potatoes.

**Table 5:** **Average longitudinal and transverse distance under different densities in the two growing seasons**.

**Density (× 10^4^ Density (plant hm^−2^)**	**Average longitudinal distance (cm)**		**Average transverse distance (cm)**	
	**Spring**	**Fall**	**Spring**	**Fall**
6	6.90c	5.42d	13.63a	10.48a
9	7.20b	5.82c	12.09b	9.58b
12	7.32b	5.90b	11.41c	8.92c
15	7.86a	5.92b	10.31d	8.44cd
18	8.05a	6.05a	10.60d	7.93d
Average	7.47	5.82	11.61	9.07

*Data are presented as means of three replicates in each treatment. Different letters in each column represent significant differences at p < 0.05*.

### Cumulative frequency of tuber number distribution under different plant densities

**Figure 1:**
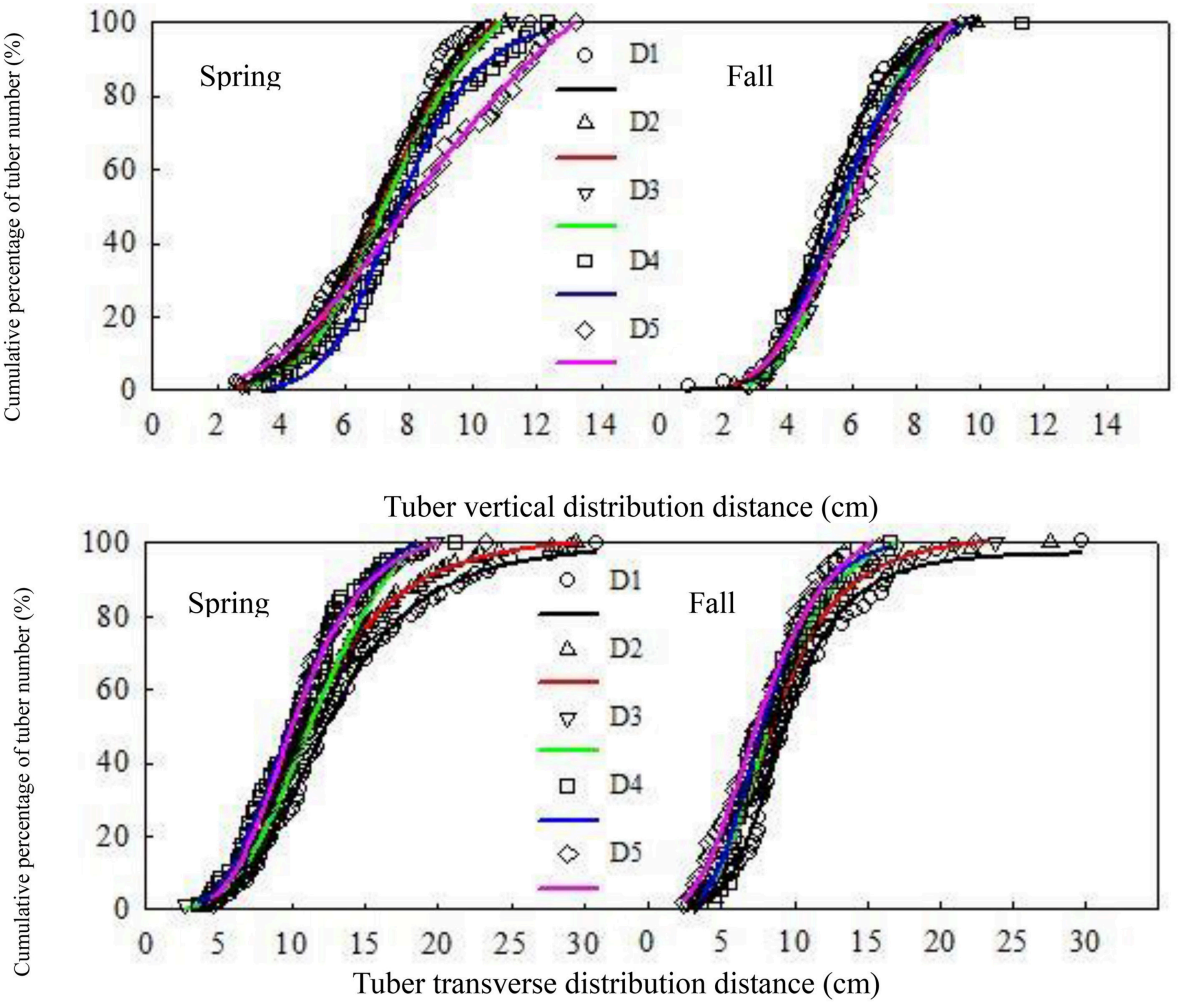
**Cumulative percentage of tuber number under different densities in the two growing seasons**. Plant densities: D1, 6 × 10^4^ strains hm^−2^; D2, 9 × 10^4^ strains hm^−2^; D3, 12 × 10^4^ strains hm^−2^; D4, 15 × 10^4^ strains hm^−2^; and D5, 18 × 10^4^ strains hm^−2^.

The vertical distribution distance increased with the increasing plant density, whereas the longitudinal distribution distance decreased (Figure 1). Moreover, the transverse distribution distance decreased significantly, and the tuber concentration increased over the two growing seasons. The longitudinal and transverse cumulative percentage (*F*) exponentially increased with the increasing distribution distance (*u*) in the two growing seasons, and the changes were in line with the following logistic Equation: *F* = *a*∕((*u*∕*c*)*b*+1) (Table 6).

**Table 6:** **Cumulative percentage equation parameter values associated with potato tuber number and equations used to determine coefficients (***R***^**2**^) under different plant densities**.

**Growing season**	**Density (× 10^4^ plant hm^−2^)**	**Tuber vertical distribution equation**				**Tuber transverse distribution equation**			
		***a***	***b***	***c***	***R*^2^**	***a***	***b***	***c***	***R*^2^**
Spring	6	128.04	−4.32	7.67	0.9894^**^	100.10	−3.93	12.34	0.9975^**^
	9	121.23	−4.67	7.69	0.9977^**^	102.49	−3.78	11.24	0.9987^**^
	12	118.49	−4.90	7.73	0.9972^**^	124.03	−3.62	12.53	0.9936^**^
	15	102.72	−6.41	7.75	0.9959^**^	111.87	−3.75	10.46	0.9928^**^
	18	146.33	−2.84	10.04	0.9960^**^	106.27	−4.22	10.34	0.9933^**^
	6	106.26	−5.23	5.39	0.9979^**^	99.49	−4.07	9.15	0.9920^**^
Fall	9	112.68	−4.46	5.94	0.9961^**^	102.10	−3.88	8.62	0.9959^**^
	12	106.90	−5.57	5.87	0.9980^**^	106.92	−3.89	8.59	0.9784^**^
	15	109.59	−4.71	5.83	0.9631^**^	103.35	−4.19	7.98	0.9796^**^
	18	129.71	−3.92	6.71	0.9943^**^	114.10	−2.94	7.84	0.9918^**^

^**^; stands for the tuber number cumulative percentage to fit for equation reached extremely significant level, respectively.

After fitting the equations to spring and fall data (Table 7), the variation coefficients of the longitudinal distribution at a 50% tuber distribution distance were 5.80 and 4.42%, respectively. At a 90% tuber vertical distribution distance, the spring and fall variation coefficients were 9.74 and 3.74%, respectively. Moreover, at a 50% transverse tuber distribution distance, the spring and fall variation coefficients were 9.11 and 8.98%, respectively, and the variation coefficients at a 90% tuber transverse distribution distance were 15.26 and 10.94%, respectively. The results clearly indicated that the influence of distribution density on the tuber horizontal distance was greater. Furthermore, the tuber distance decreased with the increasing plant density when the two growing seasons reached 50 and 90% of the transverse distribution. Compared with the highest density, the minimum density of spring and fall potato distances associated with the tuber transverse distribution of 90% were 38.81 and 29.58% greater than that of the highest density, respectively.

**Table 7:** **Potato tuber distribution distance of 50 and 90% at each plant density in the two growing seasons**.

**Growing season**	**Density (× 10^4^ plant hm^−2^)**	**50% tuber distribution distance (cm)**		**90% tuber distribution distance (cm)**	
		**Longitudinal**	**Transverse**	**Longitudinal**	**Transverse**
Spring	6	6.92	12.33	9.36	21.53
	9	7.13	11.10	9.65	18.95
	12	7.25	11.24	9.78	16.39
	15	7.69	9.88	10.52	15.25
	18	7.97	10.05	11.84	15.51
	6	5.27	9.17	7.48	15.90
Fall	9	5.65	8.53	8.09	14.46
	12	5.74	8.31	7.93	13.20
	15	5.62	7.86	8.06	12.58
	18	5.96	7.20	8.27	12.27

### Artificial neural network model of the tuber weight spatial distribution

Due to ecological factors associated with spring and fall (Table 2), the average weight per tuber differed significantly. When plant density (*x*), tuber longitudinal distribution distance (*z*), and the transverse distribution of tuber distance (*h*) were used as variables, model training, and model validation of the tuber weight (*Y*) of the artificial neural network (ANN) were established. The results indicated that the two growing seasons better responded to spatial distribution models of tuber weight at different densities. The model training and validation decision coefficients (*R*^2^) for the two growing seasons were over 0.86, and the root mean square error and mean absolute deviation were ~10 g (Table 8).

**Table 8:** **Artificial neural network model of the spatial distribution of potato tuber weight parameters during different growing seasons**.

**Growing season**	***R***^**2**^		**MRSE (g)**		**Mean absolute deviation (g)**		**Sum frequency**	
	**Train**	**Validation**	**Train**	**Validation**	**Train**	**Validation**	**Train**	**Validation**
Spring	0.8911	0.8933	11.7885	12.3637	9.6917	10.6256	100	51
Fall	0.9135	0.8677	11.3406	12.9161	9.3813	11.0060	100	50

The following equations were used to determine the spring potato tuber weight (Y) under spatial distribution models at different densities:

*Y* = −45.93*H*_1_−58.65*H*_2_+81.25*H*_3_−25.92
*H*_1_ = tanh [0.5 × (0.1051 *x* + 0.2912 *z* + 0.1131 *h* − 6.7927)]
*H*_2_ = tanh [0.5 × (0.0955 *x* − 1.0442 *z* + 0.4875 *h* − 2.1808)]
*H*_3_ = tanh [0.5 × (0.0492 *x* + 0.0580 *z* + 0.5001 *h* − 5.1615)]

The following equations were used to determine the fall potato tuber weight (*Y*) under spatial distribution models at different densities:

*Y* = −89.35*H*_1_+21.09*H*_2_+92.12*H*_3_+14.83
*H*_1_ = tanh [0.5 × (0.0433 *x* − 0.6260 *z* + 0.2673 *h* − 0.7287)]
*H*_2_ = tanh [0.5 × (−0.1541 *x* + 0.3231 *z* + 0.2202 *h* − 3.2938)]
*H*_3_ = tanh [0.5 × (0.0479 *x* − 0.2165 *z* + 0.4430 *h* − 2.9781)]
*H*_1_, *H*_2_, and *H*_3_ represented the ANNs at three different weights in the hidden layer. Using these models, different space positions under different plant densities were predicted based on potato piece weights and potato piece weight distribution ranges.

**Figure 2:**
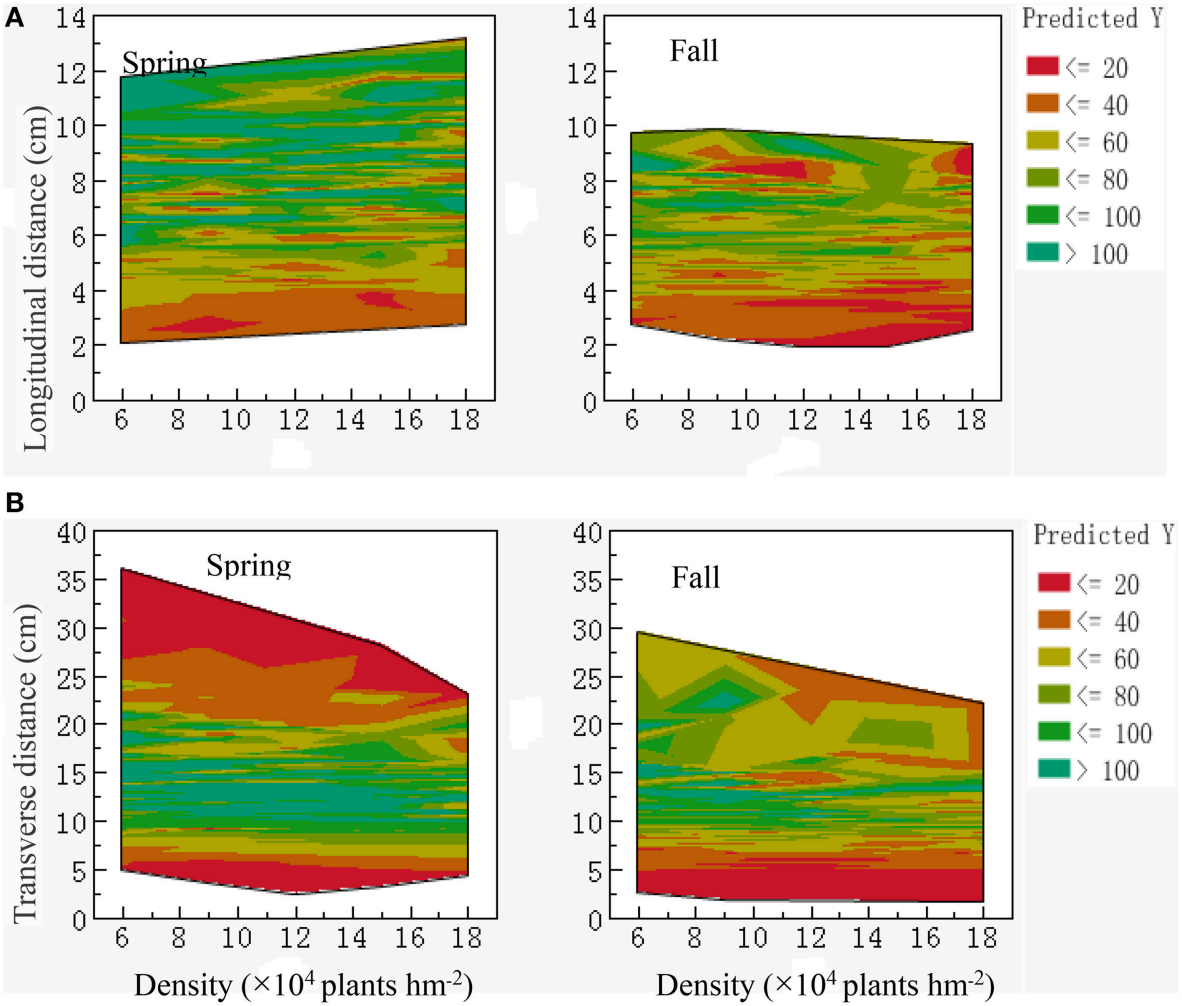
**Contour maps of potato tuber weight distribution model predictions under different plant densities**. **(A)** Longitudinal distance (cm); **(B)** Transverse distance (cm).

A contour map was constructed using model predictions to estimate the longitudinal distance (Figure 2A). At 0–4-cm depth, tubers were mainly under 40 g in both growing seasons. Tubers over 80 g were mainly concentrated at 4–6-cm depth. In vertical distances greater than 10 cm, plant density was negatively associated with tuber size in both growing seasons. Regarding the transverse distance (Figure 2B), tuber weights in both growing seasons increased with the increasing distance of transverse distribution. At 0–5-cm depth, tubers were ~20 g, whereas at 5–10-cm depth, they were 20–80 g. However, no significant differences between the various densities were identified. Furthermore, 80-g tubers exhibited a transverse distribution that differed from that of smaller tubers. In the spring, tubers greater than 80 g, which were planted at the lowest density, were mainly distributed at 12–20-cm depth. However, when planted at the highest density, the tubers were largely distributed at 10–15-cm depth. When the transverse distribution distance was greater than 20 cm, spring tubers were ~40 g and fall tubers 20–60 g.

## Discussion

Ecological conditions that mainly affect yield are light, temperature, and water, but the level of influence is different (Song and Hou, 2003; Yao et al., 2009). Previous studies have shown that the size of the potato leaf area is closely related to plant light interception rate and dry matter yield (Men and Meng-Yun, 1995) and that plant growth and material accumulation determine the crop yield. Long photoperiod negatively affects the formation, enlargement, and number of tubers (Van Dam et al., 1996; Xiao and Guo, 2010), whereas short photoperiod length reduces photosynthesis (Qin et al., 2013). Low light conditions cause a series of shade avoidance responses such reductions in plant height, internode length, and branching number (Du et al., 2013). In contrast, high light and temperature conditions promote dry matter accumulation and transportation (Deng et al., 2012). During the seedling-tuber bulking of fall potato in southern China, high temperature, and humidity conditions suppress the normal plant vegetative growth and potato tuber formation, whereas low temperature and humidity conditions (Table 2) negatively affect tuber formation and enlargement, tuber number, and yield of fall potato. The average longitudinal and transverse distribution distance of spring potatoes is higher under low rainfall and loose soil texture conditions.

The plant growth and accumulation of dry matter distribution is different under different ecological conditions as well as the influence of density on yield and its components. Increase in plant density is beneficial for improving population structure and yield (Li et al., 2010, 2011). Comparison of the growing seasons showed that the ecological factors differed greatly, and that plant growth and yield formation were not consistent. Therefore, the influence of density on yield and its components resulted in specific differences (Yao et al., 2010; Xiao, 2013). The relationship between density and yield of spring potatoes fit a convex quadratic function, whereas an increasing linear relationship was observed between fall potato yield and density. The impact of density on the average spring potato weight was greater than that observed on fall potato weight, but the influence of the average individual junction on fall potato tubers was greater than that observed on spring potato tubers.

Several parameters played major roles in determining tuber size, including photosynthetic product quantity, tuber growth, and development via the regulation of the tuber number per unit area and the average tuber weight distribution. The results indicated that plant density could significantly increase the tuber number per unit area and decrease tuber weight. Tuber number was positively correlated with the average distribution and longitudinal distance, but negatively correlated with the transverse distribution of the average distance. The correlation coefficients for spring potatoes were 0.9404^*^ and 0.9261^*^, respectively, whereas those for fall potatoes were 0.8769 and 0.8769^^**^;^, respectively. These results indicated that density could significantly influence the spatial distribution of tuber distance by regulating the tuber number. Moreover, it reduced the concentration associated with the longitudinal tuber distance, and it increased that associated with the transverse tuber distance (Figure 1).

The factors associated with the decreased rate of large and medium tubers and increased rate of small tubers were largely influenced by high plant densities (Luo, 2011; Lei et al., 2013). The number of tubers over 80 g was significantly decreased with the increasing density, and the distribution range also reduced by the establishment of tuber weight spatial distribution under different density ANN models (Seyed et al., 2014). At different planting densities, longitudinal (0–6 cm) and transverse (0–12 cm) parameters were prioritized in tubers over 80 g. Moreover, tuber weight increased with the increasing distance, and the influence of density was not immediately apparent. Tubers over 80 g were mainly distributed horizontally (12–20 cm) and vertically (6–10 cm) in space. Under high-density conditions (≥15 × 10^4^ tubers or plant hm^−2^), the transverse distribution and the tuber number ranges were significantly reduced. When the vertical distance was greater than 10 cm and the lateral distance was greater than 20 cm, tubers over 80 g were significantly reduced. Additionally, the tuber weight decreased with the increasing vertical and horizontal distances. These results illustrated that density mainly affected the tuber number and spatial distribution of tubers larger than 80 g.

## Conclusion

In conclusion, increased density significantly increased potato yield, but the degree of influence associated with different growing seasons differed slightly. Therefore, the methods used to improve yield might vary based on the growing season. These values did not differ significantly with regard to plant density. In addition, the effective control of density on tuber number (based on the number per unit area and potato tuber size) could significantly affect the longitudinal and transverse distance concentrations, Thus, density changes within a certain range could be used to regulate the spatial distribution of potato tubers, and this could be accomplished by adjusting the planting density or the mechanical harvesting parameters. This in turn would lead to the mechanization of potato production in southwestern China.

## Author contributions

SZheng accomplished the whole experiment and article. LW did the field trail. NW, LZ, and SZhou helped SZheng did the index of potato, WH provided the potato, chuanyu 117, JY supported the fund to this article and directed the article accomplished.

### Conflict of interest statement

The authors declare that the research was conducted in the absence of any commercial or financial relationships that could be construed as a potential conflict of interest.
